# In vivo anti-hyperglycemic and antioxidant potentials of ethanolic extract from *Tecomella undulata*

**DOI:** 10.1186/1758-5996-4-33

**Published:** 2012-07-06

**Authors:** Suresh Kumar, Sunil Sharma, Neeru Vasudeva, Varun Ranga

**Affiliations:** 1 Pharmacology Division, Department of Pharmaceutical Sciences, Guru Jambheshwar University of Science and Technology, Post Box: 38, Hisar, 125001, India; 2 Pharmacognosy Division, Department of Pharmaceutical Sciences, Guru Jambheshwar University of Science and Technology, Post Box: 38, Hisar, 125001, India

**Keywords:** *Tecomella undulata*, Streptozotocin, Diabetic rats, Malondialdehyde, Glycosylated hemoglobin

## Abstract

This study was undergone to evaluate the *in-vivo* anti-hyperglycemic and antioxidant potential of ethanolic extract of leaves of *Tecomella undulata* Seem. on streptozotocin-nicotinamide induced type 2 diabetic rats. Type 2 diabetes was induced by single intraperitoneal injection (i.p.) of 60 mg/kg streptozotocin, 15 minutes after the i.p administration of 110 mg/kg body weight of nicotinamide. The extract has shown significant blood glucose lowering effect in the oral glucose tolerance test (OGTT). The blood glucose level, cholesterol, glycogen contents, glycosylated hemoglobin, and antioxidant parameters (Malondialdehyde and Glutathione level) were estimated from the blood plasma by using standard kits to demonstrate the hypoglycemic and antioxidant effect in treated animals. The data showed that the extract have significant influence on the above biochemical parameters. Thus ethanolic fraction of the plant *Tecomella undulata* can be used as new candidate for antihyperglycemic and antioxidant.

## Introduction

Diabetes mellitus is a common disorder of carbohydrate, fat, and protein metabolism in which the circulating glucose concentration is increased and it causes complications such as retinopathy, microangiopathy, neuropathy and nephropathy [1]. This is a chronic incurable condition due to insulin deficiency or lesser action or both that affect 10% of the population [2]. Currently diabetes affects 150 million people worldwide and this is likely to increase to 300 million by the year 2025 [3]. It has been estimated that diabetic patients in India are expected to increase 57.2 million by the year 2025 [4]. There are a lot of synthetic derivatives which are used in the treatment of diabetes. But now days, there are increase interest in herbal agents as therapeutic treatment due to their limited adverse effects [5-7].

In India, family Bignoniaceae is represented by 21 genera and about 25 species including the non indigenous ornamental plants. The genera of Tecomella are a monotypic genus shrub/tree of the arid zone region in India. The plant *Tecomella undulata* Seem. (Bignoniaceae) is commonly known as desert teak or Rohida. The species has been identified as an important for environmental conservation in arid zones as a stabilizer of shifting sand dunes, providing shelter for wild life. It is also a very useful species for afforestation of the drier tracts due to its drought and fire resistant properties [8]. It occurs on flat and undulating areas including gentle hill slopes and sometimes also ravines and thrives very well on stabilized sand dunes, which experience extreme low and high temperature. It plays an important role in ecology; it acts as a soil binding tree by spreading a network of lateral root on the tops surface of the soil. The tree is propagated from seeds or cutting and succeeded well in drained fibrous land [9]. It is a common agro forestry tree species in the Thar Desert of Rajasthan for its higher survival rates even in extreme drought conditions [10]. It has a significant anticancer, hepatoprotective [11], analgesic [12] and antibacterial activity [13,14]. The plant contains the major constituents like tecomine, β-sitosterols, chromones glycosides, undulatosides, tecomellosides, tecosides lapachol and veratric acid [15-17]. The plant is used traditionally in the treatment of diabetes but the scientific reports are not available till date. So the work has planned to prove scientifically that the plant is used to combat this metabolic disorder for the welfare of the society.

## Materials and methods

### Drugs and chemicals

Drugs used in the present study were procured from different sources as shown: Metformin (Ranbaxy Labs, Gurgaon, India), streptozotocin, heparin (SRL, Mumbai, India), EDTA (Hi-media Lab. Pvt Ltd., Mumbai, India), n-butanol, acetic acid, n-hexane, petroleum ether, ethyl acetate, glucose standard, citric acid, sodium citrate, tris hydrochloride, buffer tablet, sodium lauryl sulphate, thiobarbituric acid, trichloroacetic acid, triton-X, glycogen, ethanol, Tween 80, carboxymethyl cellulose, Ellman’s reagent (5,5’-dithiobis-(2-nitro-benzoic acid); DTNB), sodium sulphate, methanol, pyridine, anthrone, thiourea, benzoic acid, sodium chloride (SD Fine Chem Ltd., Mumbai, India).

### Preparation of plant extract

The leaves of *Tecomella undulata* was collected from the local fields of Shekhawati area of Rajasthan (India) and identified by Dr. H. B. Singh, Head, Raw Materials Herbarium and Museum, National Institute of Science Communication and Information Resources (NISCAIR), India (NISCAIR/RHMD/Consult/-2011-12/1767/67). The leaves were dried at 40 ± 1°C, grounded into a granulated powder and defatted with petroleum ether. The ethanolic extract was obtained by extracting 2 kg of leaves powder macerated with ethanol (95%) at 25°C for 7 days followed by filtration and concentrated in water bath. All the extract was stored at temperature below 10°C and freshly prepared with 2% Tween 80 for pharmacological experiments.

### Experimental animals

Albino wistar rats (Body Weight 150-250 g) were procured from Disease Free Small Animal House, Lala Lajpat Rai Veterinary University, Hisar (Haryana), India. The rats were housed in cages (Polycarbonate cage size: 29 × 22 × 14 cm) under laboratory conditions with alternating light and dark cycle of 12 h each. The animals had free access to food and water. The animals were kept fasted 2 h before and 2 h after drug administration. The experimental protocol was approved by Institutional Animals Ethics Committee (IAEC) and animal care was taken as per the guidelines of Committee for the Purpose of Control and Supervision of Experiments on Animals (CPCSEA), Govt. of India (Registration No. 0436).

### Induction of diabetes

The type-2 diabetes mellitus (NIDDM) was induced in overnight fasted animals by a single intraperitoneal injection of 60 mg/kg streptozocin, 15 minute after the i.p. administration of 110 mg/kg nicotinamide. Hyperglycemia was confirmed by the elevated blood glucose levels determined at 72 hour then on 7^th^ day of the injection. Only rats confirmed with permanent NIDDM (Glucose level above 250 mg/dl) were used in the antidiabetic study [18].

### Experimental design

Rata were divided into five groups comprising six rats in each group after the induction of diabetes.

Group 1 Normal control rats administered 2% Tween 80

Group 2 Diabetic control rats administered 2% Tween 80

Group 3 Diabetic animals were administered metformin (100 mg/kg; p. o.)

Group 4 and 5 Diabetic animal were administered orally 200 mg/kg p.o. and 500 mg/kg p. o. ethanolic extract of *Tecomella undulata* leaves respectively

### Sample collection

#### Blood sample

The 24 h fasted animals were sacrificed by cervical decapitation on 30 th day of treatment. Trunk blood was collected in heparinized tubes and the plasma was obtained by centrifugation at 5000 rpm for 5 minutes. It was used for the determination of biochemical parameters like plasma glucose, cholesterol, malondialdehyde (MDA), reduced glutathione etc. While whole blood was used for glycosylated hemoglobin.

### Collection of organs

The rats were euthanized by using the overdose of intraperitoneal anesthesia, and tissue sample were taken for assessment of biochemical parameters.

### Estimation of plasma glucose and cholesterol

Plasma cholesterol and glucose level were measured by commercial supplied biological kit Erba Glucose Kit (GOD-POD Method) and Erba Cholesterol Kit (CHOD-PAP Method) respectively using Chem 5 Plus-V_2_ Auto-analyser (Erba Mannhein Germany) in plasma sample prepared as above. Glucose and cholesterol values were calculated as mg/dl blood sample.

### Estimation of glycosylated hemoglobin (Hb1Ac)

Glycosylated hemoglobin was measured using commercial supplied biological kit (Erba Diagnostic) in plasma sample prepared as above using Chem 5 Plus-V_2_ Auto-analyser (Erba Mannhein Germany). Values are expressed as the percent of total hemoglobin.

### Estimation of MDA level

Malondialdehyde (MDA), an index of free radical generation/lipid peroxidation, was determined as described by Okhawa *et al.* 1979 [19]. Briefly, the reaction mixture consisted of 0.2 ml of 8.1% sodium lauryl sulphate, 1.5 ml of 20% acetic acid (pH 3.5) and 1.5 ml of 0.8% aqueous solution of thiobarbituric acid added to 0.2 ml of blood plasma. The mixture was made up to 4.0 ml with distilled water and heated at 95°C for 60 min. After cooling the contents under running tap water, 5.0 ml of n-butanol and pyridine (15:1 v/v) and 1.0 ml of distilled water was added. The contents were centrifuged at about 3000 rpm for 10 min. The organic layer was separated out and its absorbance was measured at 532 nm using double beam UV-Visible spectrophotometer (Systronics 2203, Bangalore, India) against a blank. MDA values are calculated using the extinction coefficient of MDA-thiobarbituric acid complex 1.56 × 10^5^ l/mol × cm and expressed as nmol/ml [19].

### Estimation of plasma reduced glutathione level

The tissue sample (Liver 200 mg) was homogenized in 8.0 mL of 0.02 M EDTA in an ice bath. The homogenates were kept in the ice bath until used. Aliquots of 5.0 mL of the homogenates were mixed in 15.0 mL test tubes with 4.0 mL distilled water and 1.0 mL of 50% trichloroacetic acid (TCA). The tubes were centrifuged for 15 min at approximately 3000 × *g*, 2.0 mL of supernatant was mixed with 4.0 mL of 0.4 M Tris buffer pH 8.9, 0.1 mL Ellman’s reagent [5,5-dithiobis-(2-nitro-benzoic acid)] (DTNB) added and the sample shaken. The absorbance was read within 5 min of the addition of DTNB at 412 nm against a reagent blank with no homogenate. Results were expressed as μmol GSH/g tissue [20].

### Estimation of liver glycogen content

Liver glycogen estimation was done by the method as described by Seifter *et al.*[21] Immediately after excision from the animal, 1 g of the liver was dropped into a previously weighed test tube containing 3 ml of 30% potassium hydroxide solution. The weight of the liver sample was determined. The tissue was then digested by heating the tube for 20 min in boiling water bath, and following this the digest was cooled, transferred quantitatively to a 50 ml volumetric flask, and diluted to the mark with water. The contents of the flask were then thoroughly mixed and a measured portion was then further diluted with water in a second volumetric flask so as to yield a solution of glycogen of 3–30 μg/ml. Five ml aliquots of the final dilution were then pipette into Evelyn tube and the determination with anthrone was carried out. The amount of glycogen in the aliquot used was then calculated using the following equation:

(1) μg  of  glycogen  in  a liquot=100 U/1.11S

U is the optical density of unknown solution. S is the optical density of the 100 μg glucose and 1.11 is the factor determined by Morris in 1948 for the conversion of the glucose to the glycogen [21].

### Statistical analysis

The data for various biochemical parameters were evaluated by use of one- way ANOVA, followed by Dunnett test using the software Sigma-Stat 3.5. In all the tests, the criterion for statistical significance was p <0.05.

## Results

### Acute hypoglycaemic effect of *Tecomella undulata* on normoglycaemic rats

The effect of the treatment with *Tecomella undulata* leaf extract on the plasma glucose in normal fasted rats is shown in Table 1. None of the tested doses presented any significant lowering of blood glucose when compared to the control animals at different time intervals at different doses.

**Table 1:** **Acute hypoglycemic effect of *Tecomella undulata *on normoglycaemic rats**

**Treatment**	**Dose**	**Mean blood glucose concentration (mg/dl) ± S.E.M**				
		**0 hr**	**1/2 hr**	**1 hr**	**2 hr**	**4 hr**
Control	-----------	75.1 ± 3.6	97.4 ± 2.5	141 ± 2.8	111 ± 2.9	82.7 ± 2.5
Metformin	100 mg/kg	83.7 ± 2.1	78.7 ± 4.2*	105 ± 3.4**	72.3 ± 2.4**	64.4 ± 4.7**
TU Eth.	200 mg/kg	74.3 ± 2.3	98.4 ± 1.4	134 ± 5.0	91.6 ± 4.8	86.3 ± 3.7
TU Eth.	500 mg/kg	76.6 ± 4.1	98.7 ± 6.0	138.8 ± 4.4	124.3 ± 6.4	85.3 ± 2.6

Values are presented as mean ± S.E.M. n = 6 in each group. One way ANOVA followed by Dunnett’s *t* test *p < 0.05,**p < 0.01Vs. Control TU Eth.: Ethanolic extract of *Tecomella undulata* leaf.

### Acute hypoglycemic effect of *Tecomella undulata* on STZ induced diabetic rats

In the acute study of *Tecomella undulata* 200 mg/kg b.wt p.o. to STZ diabetic rats showed a fall in plasma glucose level from 385 mg/dl to 346 mg/dl at 4^th^ hour when compared to 0 days value. At a dose of 500 mg/kg b.wt orally there was a significant fall in plasma glucose level from 379 mg/dl to 339 mg/dl at 4^th^ hour (p < 0.001).

### Chronic hypoglycemic effect of *Tecomella undulata* on STZ induced diabetic rats

In the chronic study of *Tecomella undulata* 200 mg/kg b.wt orally to STZ diabetic rats for 30 days showed a fall in plasma glucose level from 383.1 mg/dl to 237 mg/dl at 30^th^ day when compared to 0 day value. At a dose of 500 mg/kg b.wt orally there was a significant fall in plasma glucose level from 376 mg/dl to 185 mg/dl at 30^th^ day (p < 0.001) (Table 2).

**Table 2:** **Acute hypoglycemic effect of *Tecomella undulata *on STZ induced diabetic rats**

**Treatment**	**Dose**	**Mean blood glucose concentration (mg/dl) ± S.E.M**				
		**0 hr**	**2 hr**	**4 hr**	**6 hr**	**24 hr**
Control	-----------	385.5 ± 12	404.8 ± 10.3	397.7 ± 5.6	379 ± 6.1	341 ± 7.1
Metformin	100 mg/kg	393.4 ± 27.2	342 ± 8.9	334 ± 7.9**	280.8 ± 6.9**	363 ± 5.2
TU Eth.	200 mg/kg	385 ± 26.4	381.1 ± 26.2	346 ± 23.4**	356 ± 29*	370 ± 2.3
TU Eth.	500 mg/kg	379.8 ± 21.8	376.2 ± 20.7	339.9 ± 22.3*	330 ± 1.6**	371 ± 9.4

Values are presented as Mean ± S.E.M.; n = 6 in each group. One way ANOVA followed by *Dunnett’s t* test, * *p* < 0.05, ** *p* < 0.01, *** *p* < 0.001 vs. control; TU Eth.: Ethanolic extract of *Tecomella undulata* leaf.

### Effect of *Tecomella undulata* in glucose loaded hyperglycaemic (OGTT) rats

**Figure 1:**
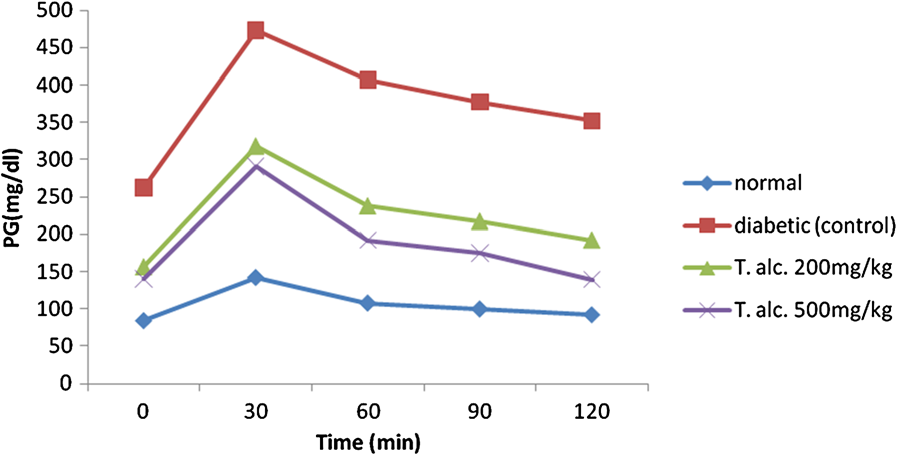
**Effect of ethanolic extract of leaves of *Tecomella undulata *on glucose loaded hyperglycaemic (OGTT) rats.** Values are presented as Mean; n = 6 in each group. PG: Plasma Glucose; T. alc: Ethanolic extract of *Tecomella undulata* leaf.

The effect of *Tecomella undulata* extract on plasma glucose level after glucose loading at 2 g/kg b.wt orally to the normal, diabetic and test animal was expressed in Figure 1. The blood glucose level rises to a maximum in 30 min after glucose loading. The extract treated groups at both 200 mg/kg and 500 mg/kg b.wt showed a significant increase in rate of clearance of glucose as compared to untreated group. The extract treated group showed a marked fall in glucose level in 30 min to 120 min interval. After 120 min the plasma glucose level returns to normal value.

### Effect of *Tecomella undulata* on glycosylated hemoglobin

**Figure 2:**
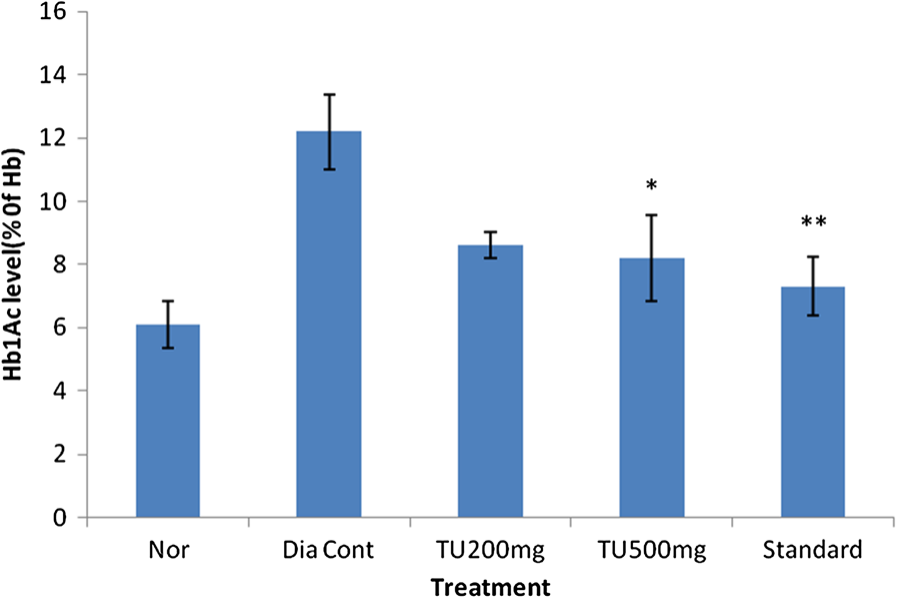
**Effect of ethanolic extract of leaves of *Tecomella undulata *on glycosylated hemoglobin (HbA1c).** Values are represented as mean ± S.E.M. one way ANOVA followed by Dunnett test; *p < 0.05, **P < 0.01 when compared to diabetic control animals. Nor: Normal Rats; HbA1c: Glycosylated Hemoglobin; TU: *Tecomella Undulata.*

The data Presented in Figure 2 indicated the effect of *Tecomella undulata* extract on glycosylated hemoglobin (HbA1c). The HbA1c data for the STZ diabetic group were significantly higher than the normal rats The Chronic administration of the standard drug significantly lowered the HbA1c in 30 days. The administration of extracts produced a similar effect when compared to the standard drug, metformin. Higher doses produced more reduction HbA1c level.

### Effect of *Tecomella undulata* on liver glycogen content

**Figure 3:**
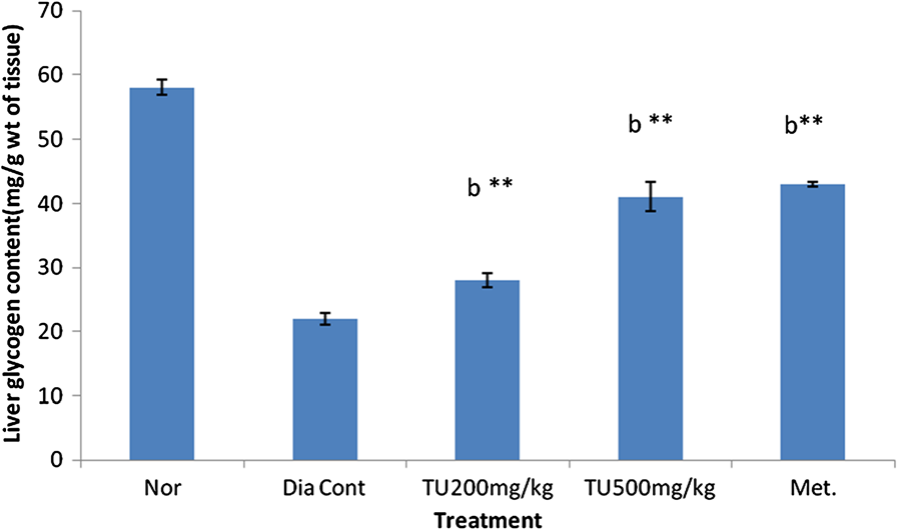
**Effect of ethanolic extract of leaves of *Tecomella undulata *on liver glycogen content.** Values are presented as mean ± S.E.M; one way ANOVA followed by Dunnett test; b P < 0.01 when compared to normal animals, **P < 0.01 when compared to diabetic control animals, Nor: Normal rats; Dia Cont: Diabetic control; Met: Metformin (100mg/kg of body wt orally); TU: *Tecomella Undulata.*

The liver glycogen level was found to be low in STZ rats when compared to normal rats *Tecomella undulata* extract at the dose level 200 mg/kg and 500 mg/kg b.wt increased the liver glycogen content significantly when compared diabetic control rats (Figure 3).

### Effect of *Tecomella undulata* on antioxidant parameters

**Figure 4:**
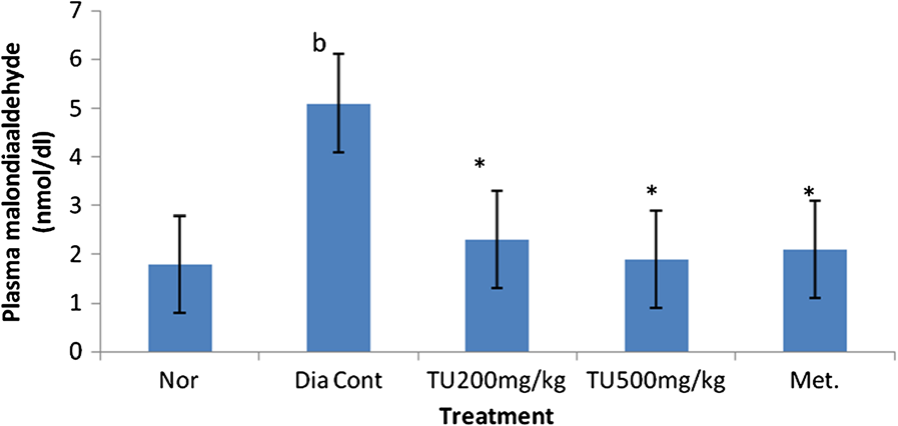
**Effect of ethanolic extract of leaves of *Tecomella undulata *on plasma malondialdehyde (MDA).** Values are presented as mean ± S.E.M; one way ANOVA followed by Dunnett test; b P < 0.01, when compared to normal group, *P < 0.05 when compared to diabetic control animals; Nor: Normal rats; Dia Cont: Diabetic control; Met: Metformin (100mg/kg of body wt orally); TU: *Tecomella Undulata.*

**Figure 5:**
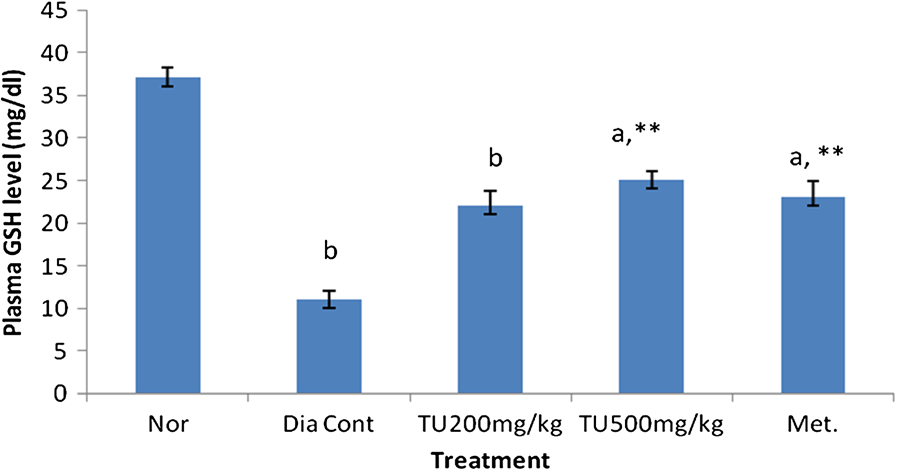
**Effect of ethanolic extract of leaves of *Tecomella undulata *on plasma glutathione (GSH) level.** Values are presented as mean ± S.E.M; one way ANOVA followed by Dunnett test; a P < 0.05, b P < 0.01 when compared to normal group; *P < 0.05, **P < 0.01 when compared to diabetic control animals; Nor: Normal rats; Dia Cont: Diabetic control; Met: Metformin (100mg/kg of body wt orally); TU: *Tecomella Undulata,* GSH: Reduced Glutathione.

The plasma level of malondialdehyde (MDA) and reduced glutathione (GSH) of normal and experimental animal in each group is shown in Figures 4 &5. Plasma MDA level in plasma was significantly increased in STZ diabetic rats compared to normal rats. Treatment of STZ diabetic rats with *Tecomella undulata* resulted in marked decrease in plasma level of MDA. The level of MDA in diabetic rats treated with *Tecomella undulata* was comparable to those in STZ diabetic rats treated with metformin (Figure 4)

The plasma glutathione level was significantly decreased in STZ rats compared to control animals (Figure 5) the level of glutathione was returned to near normal range in STZ diabetic rats treated with *Tecomella undulata* and metformin.

## Discussion

The present study was conducted to access the antihyperglycemic and antioxidant activity of *Tecomella undulata* leaves extract on STZ induced diabetic rats. STZ diabetic model is widely used for elucidation of type 2 antidiabetic activity. In the case of acute antihyperglycemic study, metformin has been selected as reference drug being its ability to modulate the insulin secretion independent of blood glucose concentration. The results presented in Table 3 indicated that *Tecomella undulata* extract produced antihyperglycemic activity in a dose dependant manner. Further at a dose of 500 mg/kg b. wt the extract produced significant antihyperglycemic activity when compared with the reference compound metformin. Here the glucose lowering activity of *Tecomella undulata* may be attributed to both pancreatic (enhancement of insulin secretion) and extra pancreatic (peripheral utilization of glucose) mechanisms. Antihyperglycemic activity at lower dose (200 mg/kg) may be due to anyone of the aforementioned mechanism.

**Table 3:** **Chronic hypoglycemic effect of *Tecomella undulata *on STZ induced diabetic rats**

**Treatment**	**Dose**	**Mean blood glucose concentration (mg/dl) ± S.E.M**			
		**1**^**st**^** day**	**10**^**th**^** day**	**20**^**th**^** day**	**30**^**th**^** day**
Control	-----------	403.8 ± 13.3	395.7 ± 7.6	373 ± 6.7	284 ± 7.2
Metformin	100 mg/kg	344 ± 9.9	251 ± 8.9***	206.8 ± 7.9***	123 ± 5.2***
TU Eth	200 mg/kg	383.1 ± 26.2	285 ± 8.6**	275 ± 7.8**	237 ± 9.9**
TU Eth	500 mg/kg	376.2 ± 20.7	263.9 ± 21.3	245 ± 23.5**	185 ± 9.5**

Values are presented as Mean ± S.E.M.; n = 6 in each group. One way ANOVA followed by *Dunnett’s t* test, * *p* < 0.05, ** *p* < 0.01, *** *p* < 0.001 vs. control; TU Eth: Ethanolic extract of *Tecomella undulata* leaf.

On treatment for 30 days, *Tecomella undulata* not only reduced the fasting blood glucose level but also improved the glucose tolerance in STZ rats. The effectiveness of *Tecomella undulata* in chronic hyperglycemia may be due to its extra pancreatic action and this is supported by improved glucose tolerance. Elevated glycosylated hemoglobin (HbAlc) was observed in the diabetic control group (12.2 ± 1.18% of Hb) when compared to normal rats (6.1 ± 0.75%) which was similar to earlier reports [22]. High glycosylated hemoglobin level indicates poor glycaemic control and responsible for the development of diabetic complications viz. vascular dysfunction, neuropathy and diabetic nephropathy. In uncontrolled and long term diabetes, there is an increased glycosylation of a number of proteins, including hemoglobin. The level of HbAlc is monitored as a reliable index of glycaemic is control in diabetes. Increase in non-enzymatic and auto-oxidative glycosylation is one of the possible mechanism linking hyperglycemia and complications of diabetes [23]. In case of diabetic rats treated with *Tecomella undulata* extract (500 mg/kg, p. o.), the HbAlc value was brought down from elevated level (12.36 ± 0.91% of Hb) to almost normal (6.24 ± 0.38; Figure 4).

Hepatic glycogen level was found to be low in diabetic rats (22 ± 1.198 mg/g wt of tissue) when compared to normal rats (58 ± 0.85 mg/g wt of tissue), which are probably due to the results of altered insulin action in diabetic state. Insulin activates the glycogen synthase system [24]. The significant increase in the glycogen level (p < 0.01) on treatment with extract (500 mg/kg) may be due to the reactivation of glycogen synthase system by *Tecomella undulata.*

*Tecomella undulata* leaves have reported to have both *in-vitro* and *in-vivo* antioxidant potential but no scientific reports are available till date in diabetic animals. Increasing evidence in both experimental and clinical studies suggested that oxidative stress plays a major role in the development and progression of both type 1 and type 2 diabetes mellitus. There has been considerable recent debate regarding the extent to which increased oxidative stress contributes towards the development of diabetic complication. The facts that the role of antioxidant compound in both protection and therapy of diabetes mellitus were also emphasized in previous scientific studies. There is an evidence that glycosylation of various protein may itself induce the generation of oxygen-derived free radicals in diabetic condition [25]. Hyperglycemia results in the generation of free radical which can exhaust antioxidant defense thereby leading to the disruption of cellular function, oxidative damage to membrane and enhance the susceptibility to lipid peroxidation [26] and diffuse from the site of tissue damage which is measured by malondialdehyde level. Fivefold increases in plasma malondialdehyde level was observed in diabetic rats (5.1 ± 0.71 nmcl/ml) compared to normal rats (1.8 ± 0.23 nmol/ml) which is similar to earlier report [27]. *Tecomella undulata* extract significantly (p < 0.05) decreased lipid peroxidation in diabetic rats at both 200 mg/kg and 500 mg/kg oral doses. The extract at 500 mg/kg p. o. dose showed marked reduction in the plasma MDA level (2.07 ± 0.50 nmol/ml) than reference antihyperglycemic drug metformin (2.47 ± 0.84 nmol/ml). It can be postulated that marked reduction in plasma MDA level is a result of both direct antioxidant action and glycaemic control.

Decrease in the level of glutathione (GSH) in diabetic rats (11.89 ± 0.987 mg/dl) compared to normal rats (36.17 ± 1.205) give evidences for altered antioxidant system during diabetes (Gupta *et al.*, 1997). Reduced glutathione has an important role in regulation of cellular redox state and therefore imbalance in reduced glutathione to oxidized glutathione is a putative indicator of cellular oxidative stress [28]. Decreased level of glutathione in plasma of n-STZ diabetic rats was partly due to its utilization by the tissue to compromise the deleterious effect of lipid peroxidation [29]. *Tecomella undulata* extract (500 mg/kg p. o.) increased the level of GSH near to normal 27.74 ± 3.68) in n-STZ diabetic rats, this may be due to its direct antioxidant properties or its increased glycaemic control. In the study, reduced glycosylated hemoglobin level and improved glutathione level in extract treated rats was observed and gives a negative correlation between GSH and HbA1c in diabetic animal as reported by Giugaliano et al., 1996, which confirmed the link between hyperglycemia and GSH depletion. Indeed, in hyperglycemic condition, glucose is preferentially used in polyol pathway which consumes NADPH necessary for GSH regeneration by the GSH-Reductase enzyme. Hyperglycemia is therefore indirectly the cause of GSH depletion. As GSH is an important antioxidant molecule, its depletion leads to the increased of oxidative stress.

The phytoconstituents responsible for the antihyperglycemic effects of *Tecomella undulata* includes flavanoids, alkaloids, diterpenes, amino acid derivatives and steroidal. Antihyperglycemic effect of Tecomella undulata in both acute and chronic hyperglycemia may be attributed to increased insulin secretion and improved insulin action (decrease insulin resistance. This study supported previous results by showing improved glucose tolerance, reduced glycosylated hemoglobin and total cholesterol in diabetic rats. Effectiveness of *Tecomella undulata* in acute hyperglycemia can be explained by insulin secreting properties of its alkaloid and terpenes contents.

## Conclusion

The present study demonstrated that the ethanolic extract of *Tecomella undulata* leaves has potential antihyperglycemic and antioxidant activity in STZ diabetic rats. The mechanism of action of antihyperglycemic action may involve improved insulin secretion or peripheral glucose utilization. Further, phytochemical and pharmacological evaluations have to be carried on the *Tecomella undulata* extract in order to identify the active principles responsible for antihyperglycemic activity and its mechanism.

## Competing interests

The authors declare that they have no competing interests.

## Authors’ contributions

SK and V carried out experimental work; biochemical analysis, statistical analysis, interpretation and discussion of results related to their part of the work. SS, NV and SK designed and planned the study; drafting and revision of the manuscript. All authors read and approved the final manuscript.

## References

[B1] Inzucchi S Baron A Porte D, Sherwin R, Baron A Classification and diagnosis of diabetes mellitus Elenberg & Rifkin’s Diabetes Mellitus 2003 New York: McGraw Hill 274

[B2] Foster DW Diabetes Mellitus Harrison’s Principles of Internal Medicine 1994 United States: McGraw Hill 1979 1981

[B3] Yohanarasimhan SN Medicinal Plants of India 2000 Tamil Nadu: Vol-ll 431 432

[B4] King H Aubert RE Herman WH Global burden of diabetes 1995-2025-prevalence, numerical estimates and projections Diabetes Care 1998 21 1414 1431 10.2337/diacare.21.9.1414 9727886

[B5] Dubey GP Dixit SP Alok S Alloxan- induced diabetes in rabbits and effect of hearbal formulation D-400 Indian J Pharmacol 1994 26 3 225 226

[B6] Kumar S Kamboj J Sharma S In vivo Anti-diabetic and Anti-oxidant potential of *Psoralea corylifolia* seeds in Streptozotocin induced type- 2 diabetic rats J Heal Sci 2011 57 3 1 11

[B7] Prince PS Menon VP Pari L Hypoglycemic activity of syzigium cumini seeds: Effect on lipid peroxidation in alloxan diabetic rats J Ethanopharmacol 1998 61 1 7 10.1016/S0378-8741(98)00002-6 9687076

[B8] Shankaranarayan KA Nanda PA Ann of Arid Zone 1963 1 174

[B9] Kirtikar KR Basu BD Indian medicinal plants 1984 2 Dehradun: International Book Distributors 1841 1842

[B10] Anonymous Genetic diversity analysis in Tecomella undulata The Biome News 2003 4 89

[B11] Khatri A Garg A Agrawal SS Evaluation of hepatoprotective activity of aerial parts of Tephrosia purpurea L. and stem bark of Tecomella undulata J Ethnopharmacol 2009 1 122 10.1016/j.jep.2008.10.043 19059328

[B12] Ahmad F Khan RA Rasheed S Preliminary screening of methanolic extract of *Celastrus paniculatus* and *Tecomella undulata* for analgesic and anti-inflammatory activities J Ethnopharmacol 1994 3 193 7934089 10.1016/0378-8741(94)90085-x

[B13] Dushyent G Bohara A Toxic effect of various plant extracts on the causal organism of typhoid fever Curr Sci 2000 78 780 781

[B14] Parekh J Jadeja D Chanda S Efficacy of aqueous and methanol extracts of some medicinal plants for potential antibacterial activity Turk J Biol 2005 29 203

[B15] Rastogi RP Mehrotra BN Compendium of Indian Medicinal Plants 2006 Volume 1- 3. New Delhi: Central Drug Research Institute, Lucknow and National Institute of Science Communication and Information Resources 711

[B16] Ambasta SP The useful plants of India 2000 New Delhi: National Institute of Science and Communication 623

[B17] Nandkarni KM Nandkarni AK Indian Materia Medica 2006 3 Mumbai: Popular Prakashan Pvt. Ltd 1197

[B18] Marudamuthu AS Leelavinothan P Effect of pterostilbene on lipids and lipid profiles in Streptozotocin – Nicotinamide induced type 2 diabetes mellitus J Appl Biomed 2008 6 31 37

[B19] Okhawa H Ohishi N Yagi K Assay for lipid peroxides in animal tissue by thiobarbituric acid reaction Anal Biochem 1979 95 351 358 10.1016/0003-2697(79)90738-3 36810

[B20] Sedlak J Lindsay RH Estimation of total protein bound and non protein bound sulfhydryl group in tissue with ellman’s reagent Anal Biochem 1968 15 192 205 4973948 10.1016/0003-2697(68)90092-4

[B21] Seifter S Dayton S Molic B Mutwzter E The estimation of glycogen with the anthrone reagent In Archive Biochem 1950 25 1 191 15401229

[B22] Koeing RJ Peterson CM Jones RL Saudek C Lehrnan M Cerami A Correlation of glucose regulation and Haemoglobin Alc in diabetes mellitus N Engl J Med 1976 295 417 10.1056/NEJM197608192950804 934240

[B23] Hall PM Cook JG Sheldon J Rutherford SM Gould BJ Glycosylated haemoglobin and glycosylated plasma protein in the diagnosis of diabetes mellitus and impaired glucose tolerance Diabetes Care 1984 7 391 10.2337/diacare.7.2.147 6734381

[B24] Kishore P Role of hepatic glycogen breakdown in defective counterregulation of hypoglycemia in intensively treated type 1 diabetes Diabetes 2006 55 3 659 10.2337/diabetes.55.03.06.db05-0849 16505228

[B25] Gupta BC Nehal M Banquer NZ Effect of experimental diabetes on the activity of hexokinase, glucose-6-phosphate dehydrogenase and catecholamine in rats erythrocytes of different ages End J Exp Biol 1997 35 92 9418381

[B26] Giuglino D Ceriello A Paslisso G Oxidative stress and diabetes vascular complications Diabetes Care 1996 19 257 10.2337/diacare.19.3.257 8742574

[B27] Kwiatowsha S Piasecka GG Zieba M Pitrowski W Wowak D Increased plasma concentration of conjugated dienes and malindialdehyde in patient with pulmonary tuberculosis Respir Med 1999 93 272 10.1016/S0954-6111(99)90024-0 10464892

[B28] Exner R Wessner B Manhart N Roth E Therapeutic potential of glutathione Wien Klin Wochenschr 2000 112 610 11008322

[B29] Anuradha CV Selvan R Effect of oral methionine on tissue lipid peroxidation and antioxidant in alloxan induced diabetic rats Nutr Biochem 1993 4 4 211 10.1016/0955-2863(90)90027-i 15539186

